# Zebrafish Congenital Heart Disease Models: Opportunities and Challenges

**DOI:** 10.3390/ijms25115943

**Published:** 2024-05-29

**Authors:** Dixuan Yang, Zhenjie Jian, Changfa Tang, Zhanglin Chen, Zuoqiong Zhou, Lan Zheng, Xiyang Peng

**Affiliations:** State Key Laboratory of Developmental Biology of Freshwater Fish, Key Laboratory of Physical Fitness and Exercise Rehabilitation of Hunan Province, College of Physical Education, Hunan Normal University, Changsha 410000, China; 202220152984@hunnu.edu.cn (D.Y.); 202030172020@hunnu.edu.cn (Z.J.); changfatang@hunnu.edu.cn (C.T.); zhanglinchen@hunnu.edu.cn (Z.C.); zhouzuoqiong@hunnu.edu.cn (Z.Z.)

**Keywords:** zebrafish model, congenital heart defects, single-defect heart disease, heart disease syndrome

## Abstract

Congenital heart defects (CHDs) are common human birth defects. Genetic mutations potentially cause the exhibition of various pathological phenotypes associated with CHDs, occurring alone or as part of certain syndromes. Zebrafish, a model organism with a strong molecular conservation similar to humans, is commonly used in studies on cardiovascular diseases owing to its advantageous features, such as a similarity to human electrophysiology, transparent embryos and larvae for observation, and suitability for forward and reverse genetics technology, to create various economical and easily controlled zebrafish CHD models. In this review, we outline the pros and cons of zebrafish CHD models created by genetic mutations associated with single defects and syndromes and the underlying pathogenic mechanism of CHDs discovered in these models. The challenges of zebrafish CHD models generated through gene editing are also discussed, since the cardiac phenotypes resulting from a single-candidate pathological gene mutation in zebrafish might not mirror the corresponding human phenotypes. The comprehensive review of these zebrafish CHD models will facilitate the understanding of the pathogenic mechanisms of CHDs and offer new opportunities for their treatments and intervention strategies.

## 1. Introduction

Heart disease is a significant global cause of mortality and reduced life expectancy, with the current global prevalence of congenital heart defects (CHDs) being 9.410%, which is lower than that in China (4.905%). CHDs are structural abnormalities occurring during embryonic and neonatal heart development, accounting for approximately 30% of cardiac cases in infancy, and mortality rates are associated with the severity of these defects [1]. These defects can manifest in various heart parts, such as the heart lining, the septum between the atria and ventricles, the valves, and major arteries and veins [2]. CHDs exhibit variability in their prevalence, severity, and affected cardiac tissues and can occur alone or as part of syndromic diseases impacting multiple organs [3]. Animal models, particularly the zebrafish, have become significant in investigating the pathogenesis of CHDs, owing to their small size, high reproduction rate, rapid growth cycle, and genetic similarity to humans, with 82% of related genes having orthologous genes in humans [4]. Over the last two decades, zebrafish models have been extensively used in studies involving heart development and cardiovascular diseases [5]. Researchers have effectively used gene editing techniques to establish numerous zebrafish CHD models, enabling the verification of genome-wide association study data in patients with cardiovascular disease and facilitating clinical treatment investigations [6]. In this context, this review evaluated the application of zebrafish in CHD studies, offering insights for further leveraging this model to explore CHD pathogenesis, clinical diagnosis, and treatment.

## 2. Relevant Sections

In this section, we discussed the advantages and disadvantages of using zebrafish as a model for CHDs in vertebrates, as well as the early development of the zebrafish heart. We also summarized the former point into a table (Table 1).

### 2.1. Advantages of Zebrafish as a Model of Vertebrate CHDs

#### 2.1.1. Cardiovascular System Has Physiologically Advantageous Characteristics

Zebrafish is significant among model organisms owing to its similarity in heart rate to that of humans. An adult zebrafish exhibits clear P, QRS, and T waves on electrocardiogram analysis [7], closely resembling human cardiac electrophysiology [8,9,10]. In contrast, traditional animal models, such as mice, excessively rely on the cardiovascular system for obtaining oxygen during early embryonic development. When CHDs occur in mouse embryos, they can rapidly cause poor overall health or even death, with limited regenerative capacity post-birth, occurring only within the first 7 days [11,12,13]. Conversely, zebrafish embryos can survive for a period even without a fully functional cardiovascular system, obtaining oxygen through passive diffusion. Notably, the zebrafish heart can fully regenerate after injury [14,15,16,17]. Moreover, the zebrafish embryo’s heart develops considerably faster than that of other model animals, beating for just one day as opposed to a week for mice [5,18]. The zebrafish has six pairs of aortic arch arteries, unlike mammals that possess only five pairs. Nonetheless, the formation and pattern of aortic arch arteries in zebrafish closely resemble that in mammals [19,20,21]. These unique characteristics make zebrafish an excellent model for studying various aspects of cardiovascular development, CHDs, and cardiac regeneration mechanisms.

#### 2.1.2. Imaging Technology Is Mature

Optogenetics involves measuring and manipulating cellular activity using genetically encoded light-sensitive proteins [22,23]. In terms of cardiology, optogenetics has wide applications [24]. With their optical transparency, zebrafish embryos and larvae enable the visualization of morphological changes in the cardiovascular system during development through transmitted light or fluorescence imaging techniques [20]. Additionally, two-photon microscopy facilitates the imaging and analysis of cardiac functions in zebrafish [25]. Leveraging transgenic zebrafish technology, researchers can simultaneously observe instantaneous cardiac calcium changes and contractions in juvenile fish [26,27]. Zebrafish have become significant in cardiac optogenetic studies with technological advancements. For example, researchers use optical tools and transgenic zebrafish possessing light-gated ion channels (rhodopsin) to locate pacemaker points in the heart and apply specific light-stimulation methods. This approach enables the optical control of the heart rate, the simulation of various heart disease states (such as tachycardia and bradycardia), and the elucidation of organ function emergence during development [28]. For example, researchers utilized optogenetics to study the impact of the zebrafish Popeye domain containing a gene 2 knockout. This led to irregular atrial and ventricular activities, atrioventricular node conduction failure, and varying degrees of atrioventricular blockage [29]. With the continuous improvement of optogenetic techniques [24], research into the pathological or physiological structure and function of the heart using zebrafish has become feasible.

#### 2.1.3. Gene Editing Tools Are Mature

Comparing the human reference genome with the zebrafish genome revealed that approximately 70% of human genes have at least one clear zebrafish ortholog [4]. Recent studies have further revealed a more comprehensive map of zebrafish genetic data, building upon the known genome sequence of zebrafish [30]. Forward and reverse genetics methods have mature application technologies in zebrafish embryos. The forward genetics method involves inducing random mutations through radiation, chemical treatment, or the insertion of exogenous DNA to obtain a strain with a stable pathological phenotype, enabling the exploration of the genetic basis of diseases. For example, forward genetics methods have been pivotal in screening and identifying mutations that cause valve defects and aortic coarctation [31,32]. Conversely, reverse genetics involves using gene editing technology to directionally alter previously identified genes and analyze the consequential effects of these genetic changes on organisms [33]. An example of this approach includes using the clustered regularly interspaced short palindromic repeats (CRISPR)/Cas9 technology to develop a *heg1* knockout zebrafish strain, revealing the regulatory role of *heg1* in heart failure and thrombosis and its potential application in cardiovascular drug screening [34]. The high degree of genetic conservation, ease of genetic manipulation, external development, and availability of large numbers of embryos daily accelerate research on CHDs, making zebrafish an excellent model for studying CHDs. In the Section 3 below, we focus on reviewing the use of gene editing to model CHDs in zebrafish, which will contribute to our understanding of the mechanism of CHDs.

### 2.2. Disadvantages of Zebrafish as a Model of Vertebrate CHDs

The heart is the first organ to develop and function in the embryo of zebrafish. In contrast to those in mammals, the coronary vessels in zebrafish develop within the first few weeks post-fertilization, and the myocardial trabeculae do not undergo compaction, remaining as permanent structures [5,35]. In addition, some limitations stem from differences between zebrafish and mammalian anatomy and physiology; zebrafish have only two wall chambers, lack a pulmonary circulation, and lack a conduction system with specialized Purkinje fibers, which limits the generation of models of septal defects or conduction system diseases. Furthermore, in fish, diastolic ventricular filling is primarily determined by atrial contraction, unlike the central venous pressure in humans [36].

Most of the calcium responsible for ventricular cardiomyocyte contraction in humans originates from intracellular sarcoplasmic reticulum stores; however, in zebrafish sarcoplasmic reticulum, calcium release is limited [37]. The lack of sarcoplasmic T-tubules is less dependent on sarcoplasmic reticulum calcium cycling and more dependent on sarcoplasmic T-type calcium currents [38]. The majority of calcium sources are extracellular and enter primarily through sarcoplasmic T-type calcium channels [38,39]. In contrast, in humans, the calcium-induced generation of sarcolemmal calcium currents occurs through L-type calcium channels, while T-type calcium channels are significantly absent [40]. In the healthy mammalian heart, the force–frequency relationship is positive; that is, the force of myocardial contraction increases with increasing heart rate [41]. These differ from observations in zebrafish [42]. These aspects need to be considered when using zebrafish to model human myocardial contractility-related diseases. Despite the similarity of the electrophysiological basis of the heart between zebrafish and humans, as previously recounted, there are still differences in the flow of ions, that is, the depolarizing flow of sodium ions, calcium ions, and the repolarizing flow of potassium ions [39]. Therefore, a better understanding of zebrafish cardiac electrophysiology and its limitations is needed to model diseases related to human cardiac electrophysiology. Notably, there has been successful modeling of human genetic repolarization disorders using zebrafish [7,43,44]. Notably, this may be a model of choice for cardiac electrophysiology associated with abnormal repolarization but may be less suitable for the study of depolarization disorders or calcium-regulated arrhythmias [45]. Moreover, the adult zebrafish heart contains a small number of fibroblasts [46]. While various stimuli trigger fibrosis in mammals, they do not produce similar results in zebrafish, limiting our capacity to mimic specific fibrotic pathologies [47,48].

During teleost evolution, the zebrafish genome underwent a duplication event, leading to the existence of two copies of several genes that typically perform redundant functions [49,50]. This genomic duplication poses a challenge in generating mutants, as it necessitates the simultaneous knockdown of both copies of the genes. Compared to mammalian models, the availability of antibodies and other reagents for zebrafish is relatively limited. In addition, some zebrafish genes have no homologs of known human genes but exercise functions similar to other genes. This should be remembered when researching a specific gene related to CHDs in zebrafish.

### 2.3. Early Heart Development in Zebrafish

**Figure 1:**
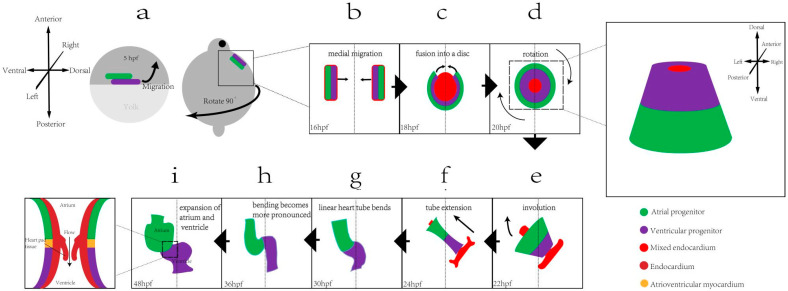
Zebrafish heart development process. (**a**) Cardiac precursor cells are initially on the ventrolateral side, while ventricular precursor cells are on the dorsal side and edge of atrial precursor cells. Endocardial precursor cells are on the medial side of both precursor cells; (**b**) cardiac precursor cells migrate medially; (**c**) intracardiac mesangial cell groups form the cardiac disc at the midline, and the ventricular and atrial precursor cell groups migrate toward the midline; (**d**) the myocardium begins to fuse from the posterior portion of the cardiac disc formed by the endocardium and continues to surround the endocardium to form the cardiac cone. For the ventricular anterior, the somatic cell group is at the tip, and the atrial precursor cell group is at the bottom; (**e**) the cardiac cone rotates and tilts for the atrial precursor cells to be located to the left of the ventricular precursor cells, and the endocardium and cardiomyocytes fuse; (**f**) the cardiac cone extends for the atrial precursor cell group to be located to the left and in front of the ventricular precursor cell group; (**g**) the heart tube undergoes circularization to form a slightly “S”-shaped tube, and the atrial and ventricular cavities are formed; (**h**) the heart undergoes circularization to form a morphology; and (**i**) the chamber expands, and endocardial cells and the matrix form heart cushion tissue, which form a valve and perform valve function.

During zebrafish embryonic development, the early heart originates from the ventrolateral cardiogenic zone of embryos at 3–4 h post-fertilization (hpf). By 5 hpf, atrial, ventricular, and endocardial progenitor cells cluster in the ventrolateral region before migrating toward the midline and dorsal side. In this migration process, the cells reach both sides of the midline by 16 hpf. Subsequently, at 20 hpf, the precursor cells on both sides gradually coalesce to form the cardiac cone, transitioning into the cardiac tube. By 24 hpf, the atrial cell population is distinctly positioned to the left and in front of the ventricular cell population. Visible atrial and ventricular cavities begin to form at 30 hpf. At 36 hpf, the embryonic heart adopts a circular morphology, signaling the progressive differentiation and specialization of the heart tube regions. Simultaneously, the head-end develops a primitive outflow tract, and endocardial cells start to form at the atrioventricular intersection, with matrix materials moving closer to the cardiac cushion tissue. By 48 hpf, the endocardial cushion tissue matures into a functional valve. Figure 1 shows the visual representation of these developmental stages from a to i.

## 3. Discussion

Heart defects result from an intricate interplay of genetic and environmental factors. During heart development, any slight mistake may lead to deformity or dysfunction. Mutations can affect cardiac development alone or produce a series of defects, including developmental syndromes. We discuss the genes related to single-defects and syndromes (Table 2) and highlight that the zebrafish embryo serves as a valuable model for studying the impact of teratogens on heart development.

### 3.1. Zebrafish Single-Defect CHD Model

#### 3.1.1. Heart Chamber Size Defects

During the process of chamber formation, an abnormal expression or mutations of certain chamber development-related genes, such as nuclear receptor subfamily 2 group F member 2 (*NR2F2*), may cause human CHDs. Proper cardiac chamber size and proportions are essential for effective cardiac function in vertebrates, as blood circulation relies on the continuous contraction of the atria and ventricles [79,80,81]. The two-chamber heart of zebrafish provides a simpler model for studying mechanisms related to chamber size regulation compared with the four-chamber hearts of vertebrates [82,83,84,85]. Using zebrafish models, previous studies have investigated genes and transcription factors involved in regulating chamber size.

NR2F proteins, such as NR2F1 and NR2F2, are highly conserved solitary nuclear receptors. NR2F1 is crucial in neurodevelopment, and NR2F2 is implicated in cardiac genesis [86]. The expression levels of both proteins overlap in the atrial cardiomyocytes of the heart [87,88,89,90]. NR2F2 has been particularly highlighted as being associated with CHDs [79]. Notably, it has been shown that, in zebrafish, Nr2f1a promotes atrial development and differentiation at a level equivalent to NR2F2 in mammals [51]. The atrium of *nr2f1a* mutants was observed to be smaller than that of the wild-type zebrafish, indicating a potential regulatory role of NR2F1a in atrial size and development through bone morphogenetic protein (Bmp) signaling, as revealed by a recent study [51]. This study introduces novel avenues for investigating the etiology of CHDs associated with atrial size.

Semaphorins are a large family of secreted or membrane-associated glycoproteins [91]. Among them, class 3 semaphorins (Sema3s) are used as guidance signals for cardiovascular development [92,93]. Sema3 signaling is mediated through the plexin receptor and its coreceptors, neuropilins [91]. In previous studies, a *sema3fb* knockout zebrafish model was established to investigate the molecular mechanism of chamber-specific cardiomyocyte differentiation [52]. The findings revealed that at 3 hpf, both cardiac chambers of the *sema3fb* knockout fish were smaller than those of the wild-type, with the ventricular differentiation process remaining unaffected by Sema3fb deletion; however, the cardiomyocytes in the atria were smaller than their wild-type counterparts. This discrepancy may be caused by a disruption in the expression of specific differentiation genes [52]. The secretion of Sema3 signals by cardiomyocytes potentially facilitates the establishment of a boundary between the ventricle and the atrium via spatially specific regulatory signals. This activity is essential for the normal chamber development to its appropriate size [52]. Studies involving zebrafish models have revealed an association between CHDs and aberrations in Sema3 signaling, offering valuable insights for the clinical understanding, diagnosis, and treatment of CHD-related conditions [94,95].

#### 3.1.2. Left and Right Asymmetry (Looping Defects)

Heart development is significantly influenced by left–right pattern disorders, with deviations in this pattern potentially causing heart defects [96]. An illustration of this is the development of abnormal asymmetry in the heart, causing defects in cardiac circulation and leading to CHDs during embryonic development, such as left or right atrial isomerism [97]. Zebrafish, similar to vertebrates, such as humans, mice, and chickens, undergo heart development guided by a left–right asymmetric pattern with a comparable anatomy and molecular mechanisms [98,99]. Notably, the various stages of cardiac asymmetry, commencing from the formation of the heart field to the heart tube and looping, are observable through microscopic examination [100]. Researchers have meticulously investigated the distinctive stages of asymmetric cardiac development through the zebrafish model, scrutinizing the associated events, processes, and molecular mechanisms.

The asymmetric development of the heart in zebrafish starts from the Kupffer’s vesicle (KV) [101]. This is a transient structure that marks the first break in the left–right symmetry within the embryo [102]. Within the KV, cilia act as mechanical sensors of shear force, generating a directional liquid flow known as the nodal flow; the perception of mechanical forces by the cilia is pivotal in establishing left–right patterning, and *wnt3a* and *8a* modulate cilia generation [56,101,103]. Polycystin-2 (PKD2) is crucial for intraciliary calcium oscillations within the left–right organizer. These mechanical stimuli, transmitted through ciliary structures, influence calcium transients, regulating the expression of vital asymmetric molecules [55,103,104]. However, *curly up* (*cup*) affects the gene homologous to zebrafish *pkd2*, which encodes a Ca^2+^-activated non-specific cation channel Pkd2 [55], and *southpaw* (*spaw*) is an early asymmetrically expressed gene around the KV [54]. During the 4–6 somite stage, Spaw is symmetrically expressed around the KV and gradually expands toward the left lateral plate mesoderm (LPM) from the 10–12 somite stage; this asymmetric expression depends on the leftward nodal flow within the KV [105]. *spaw* expression is regulated by *left–right determination factor 1* (*lefty1*), Bmp signaling, and *dand5*. Bmp signaling can modulate *spaw* expression through *lefty1*, influencing *spaw* expression in the LPM, while inducing *lefty1* expression in the midline [106], and *lefty1* should maintain a specific ratio with *spaw* expression [107,108,109]. Furthermore, a localized Bmp protein source induces heart tube localization, whereas the asymmetric expression of hyaluronan synthase 2 affects the correct guidance of locally expressed Bmp proteins to the heart tube [57].

The characteristic feature of lateral defects is the left–right patterning defect during embryonic development. A study used exome sequencing to screen for candidate genes in 70 patients with CHDs and lateral defects, identifying candidate genes *TRIP11*, *DNHD1*, *CFAP74*, and *EGR4* [110]. Owing to the rapid development, ease of operation, and highly conserved left–right patterning process across vertebrate species, the zebrafish was selected for analyzing candidate genes. The knockdown of zebrafish *trip11*, *dnhd1*, and *cfap74* results in significant cardiac looping abnormalities, a disturbed expression of spaw and related genes (*lefty2* and *pitx2*), and the altered formation and function of KV cilia during embryonic development, respectively. The left–right asymmetric defects of these mutant hearts can be effectively rescued by introducing the corresponding candidate mRNAs [110]. Cilia are crucial for regulating left and right patterning, and defects in genes related to their function can cause abnormal cardiac development. Therefore, zebrafish models are vital in investigating the specific roles of these genes in left–right patterning to understand CHDs resulting from human gene variations and are essential for CHD diagnosis and treatment.

#### 3.1.3. Atrioventricular Valve Defects

Heart valves are crucial in maintaining the one-way blood flow within the heart. Defects, malformations, or aberrant expression of related genes during valve development are common factors contributing to CHDs, such as bicuspid aortic valve and mitral valve prolapse [111,112,113]. Therefore, understanding the developmental processes and molecular mechanisms underlying heart valve formation is crucial for developing effective treatment methods and preventive measures. The zebrafish is a valuable model for investigating early valve development because its heart can be visualized in vivo during embryonic stages with single-cell resolution [25,114]. The formation of heart valves involves collaboration between endocardial cells and the extracellular matrix [115,116,117]. Endocardial cells are responsive to stimuli from blood flow, activating Krüppel-like factor 2a (Klf2a)-mediated mechanosensitive channels. The disruption of these mechanically sensitive channels can impact the valve morphology [118]. *klf2a* knockdown in zebrafish embryos results in underdeveloped valves, usually lacking leaflets altogether [58]. Moreover, Klf2a expression is influenced by the PKD family, which is crucial in valve formation through its mediation by Camk2g [59,119]. Mutant larvae deficient in *pkd1a* exhibit an increased incidence of retrograde blood flow at 78 hpf, with only 66% of surviving larvae displaying elongated upper leaflets, a lower rate than that of a normal development [59].

In human patients with valvular heart disease, the expression of troponin I type 1 (*TNNI1*) is inhibited or inactivated [120]. The loss of *tnni1* inhibits the myocardial Wnt signaling pathway, causing defects in atrioventricular valve development. *tnni1b* overexpression in homozygous zebrafish embryos can partially resolve valve development defects. When *tnni1b* (a homolog of human *TNNI1*, a structural and regulatory protein involved in cardiac contraction) is knocked out in zebrafish, phenotypes, such as a slowed heart rate, lack of valve leaflets, and cardiac tube malformations, occur. Additionally, all homozygous embryos died within 1 week [84]. The expression levels of other sarcomeric genes, such as *ctnnt*, *ctnnc*, *myl7*, *myh7*, and *myh6*, remain unaffected, further confirming that abnormalities in TNNI1b contribute to the observed cardiac phenotype in the mutants [60]. However, further studies are required to determine the precise mechanism by which TNNI1b influences the myocardial Wnt signaling pathway.

*NFATC1*, a nuclear factor-κB-related transcription factor, has been associated with congenital heart valve septal defects in humans [121,122,123]. Zebrafish studies have revealed that *nfatc1* deletion results in heart valve defects, and *nfatc1* knockout mutants display enlarged atria compared with wild-type fish [61]. This atrial enlargement resembles the condition observed in patients with mitral regurgitation [62], and nfatc1 knockout zebrafish exhibit retrograde blood flow in the atrioventricular canal [61]. *nfatc1* deficiency may cause a diminished recruitment and proliferation of valve interstitial cell (VIC) precursors, causing VIC defects and disturbances in valve extracellular matrix organization [61]. *nfatc1*-mediated regulation is crucial for early VIC establishment, which is essential in valve development during the embryonic stage and subsequent normal valve function in adulthood.

#### 3.1.4. Outflow Tract Stenosis

Neural crest cells are principal cell sources in the development of the cardiac outflow tract (OFT) [124], a critical component for proper cardiac function. Their migration and differentiation within the OFT region are crucial in cardiac development. However, abnormalities arising in the OFT during development can cause CHDs, which are prevalent among approximately 30% of cases [125,126]. Consequently, understanding the role of neural crest cells in OFT development is essential for comprehending CHD pathogenesis and exploring potential therapeutic interventions.

Cardiac neural crest cells are derived from a cell population originating from the cranial part of the neural tube [127]. Tumor necrosis factor receptor-associated factor 7 (*TRAF7*), a member of the multifunctional TRAF family, exhibits high expression levels in the neural crest and its derivatives, particularly in the cardiac OFT [63]. In zebrafish models, *traf7* knockout causes an increased probability of abnormal embryonic heart tube displacement, accompanied by noticeable pericardial edemas, irregular cardiac looping, and craniofacial anomalies [63]. While no OFT-specific abnormalities have been documented, the migration ability of neural crest cells to form OFT pads underscores their potential significance in this process [127]. Additionally, the significant reduction in the expression of the neural crest marker (*sox10*) in zebrafish embryos after *traf7* knockout indicates a possible association with the observed OFT defective phenotype [63]. Therefore, further investigations into the OFT defective phenotype in *traf7* knockout zebrafish are required.

During embryonic development, the neural crest is regulated by p21-activated protein kinase 1 (Pak1). This study demonstrated that *pak1* knockout in zebrafish embryos causes defects in neural crest development and, subsequently, blockage in the OFT. This finding highlights the significance of the Pak/Mek/Erk/Gata6 signaling pathway in normal neural crest development. Gata6 signaling activation via extracellular regulated protein kinases (Erk) is reportedly a central mechanism in this process [64]. Notably, *GATA6* mutations in humans have been associated with various CHDs, including OFT malformations [128]. This association underscores the significance of understanding the molecular pathways involved in neural crest development for preventing and treating CHDs.

Studies revealed that activin A receptor-like type 1 (Alk1), a transmembrane serine/threonine receptor kinase belonging to the TGF-β family and encoded by *ACVRL1*, is related to the occurrence of CHDs [129,130]. *acvrl1* mutations have been shown to affect zebrafish OFT morphology. While applying the zebrafish model, a study suggested a novel perspective on OFT development: biological signals generated by fluid forces can stimulate endocardial proliferation and an *acvrl1*-dependent increase in endothelial cells, contributing to the formation of the OFT cavity with a specific endocardial thickness [65]. In zebrafish embryos where Acvrl1 is knocked down, OFT endocardial cells fail to accumulate normally at 51 hpf, causing the narrowing of the OFT cavity [65]. This investigation introduces new insights for evaluating the etiology of CHDs associated with abnormal OFT morphology.

#### 3.1.5. Tetralogy of Fallot (TOF)

The most common type of cyanotic CHD is TOF, with an incidence rate of 0.34 per 1000 live births [131]. TOF can occur in the setting of additional non-cardiac anomalies or in isolation [132]. TOF is characterized anatomically by four structural defects, namely ventricular septal defects, right OFT/pulmonary artery stenosis, an overriding aorta, and right ventricular hypertrophy [133]. A vital clinical indicator for diagnosing TOF is the degree of right ventricular outflow tract (RVOT) stenosis [134].

The first genetic cause of TOF identified was *NKX2.5* mutation, a crucial transcription factor that controls heart development [135,136]. Similarly, *GATA4* mutation can hinder its interaction with *NKX2.5*, contributing to TOF [137]. Blood vessel epicardial substance (BVES), also known as POPDC1, is a highly evolutionarily conserved membrane protein that is highly expressed in the adult vertebrate heart [138,139]. Mutations or reduced *BVES* gene functioning are found in patients with non-syndromic TOF and heart failure [140,141,142]. In mice, *Bves* is expressed in the developing cardiomyocytes and coronary endothelial cells and in the conduction system in adult mice [143,144,145]. A study has found that the rs2275289 (p.R129W) single nucleotide polymorphism in BVES reduces its own expression and likely decreases the expression of *NKX2.5* and *GATA4* [141], and in studies involving *bves* knockdown zebrafish, abnormal annularization and ventricular OFT stenosis were observed, in addition to reduced *nkx2.5* and *gata4* expression levels, causing the manifestation of TOF, a congenital heart disease [66]. Therefore, further exploration of the connection and detailed regulatory mechanisms between the downregulation of *BVES* and the occurrence of RVOT stenosis in patients with TOF is required. Another recent study highlighted WD repeat domain 62 (*WDR62*) as a novel susceptibility gene associated with CHDs through sequencing analysis in a large number of human patients with CHDs, with a relatively high mutation frequency associated with TOF [67]. Subsequently, it was demonstrated that *wdr62* was abundantly expressed in the zebrafish heart through whole-mount in situ hybridization. The knockdown of *wdr62* by morpholino in zebrafish resulted in heart defects in 80% of zebrafish, including abnormal cardiac looping, narrowed chambers, thin chamber walls, impaired OFT rotation, and TOF-related defects, as evidenced in histological sections. Insights from this study revealed the correlation between *WDR62* and CHDs, indicating that *WDR62* could influence cardiac development by affecting spindle assembly and the cell cycle of cardiomyocytes [67]. While zebrafish hearts have a relatively simple two-chamber structure and generate lower pressures than those of humans, the aberrant annularization of fish embryos observed in *bves* and *wdr62* knockdown zebrafishes, with the former also exhibiting OFT stenosis, implies potential limitations in fully recapitulating all four classic TOF phenotypes [66,67]. Malformations in the OFT during embryonic development, similar to those observed in human patients, can trigger the emergence of the other three structural defects, even if all phenotypes are not present [146,147,148]. Given the critical role of OFT development in the pathogenesis of TOF, emphasizing abnormalities in this process through the zebrafish model can provide valuable insights into the pathophysiology of human TOF.

### 3.2. Zebrafish CHD-Related Syndrome Model

#### 3.2.1. CHARGE Syndrome

CHARGE syndrome (Coloboma, heart defects, Atresia of the choanae, retarded growth and mental development, genital anomalies, and Ear malformations), an autosomal dominant genetic disease, occurs at a frequency of 1 in 8500 to 1 in 15,000 live births worldwide [149,150]. Among individuals affected by CHARGE syndrome, approximately 80% will develop CHD [151]. These cardiac abnormalities can manifest as OFT defects, patent ductus arteriosus, atrioventricular septal defects, and aortic arch abnormalities [152]. Notably, *CHD7* mutations are identified in approximately two-thirds of patients with CHARGE syndrome, and such mutations have been detected in some individuals with isolated CHDs [152,153,154]. This suggests that *CHD7* and its downstream genes are possibly crucial in the development of cardiac defects in CHARGE syndrome [155]. *Chd7* heterozygous mice show various signs of CHARGE syndrome, including growth retardation and severe head bobbing, destruction of the lateral semicircular canals, septal defects, and genital abnormalities [156,157]. In contrast, *Chd7* heterozygous mice only live until 10.5 days after birth, making it impossible to study the overall function of *CHD7* and drug screening [154,156]. The recognized causes of death include growth retardation, axial rotation failure, peri-cardial swelling, and tail structure formation failure [158]. Zebrafish exhibit a lower tolerance to cardiovascular defects than other models, and *chd7* mutants display craniofacial anomalies, cardiac defects, gastrointestinal narrowing, and cranial nerve defects [21,159,160,161]. This is more advantageous for studying the overall function of CHD7 and cardiovascular-related diseases. Therefore, the zebrafish model is considered more suitable for studying the overall function of *chd7* and cardiovascular-related diseases than other models with a lower tolerance to cardiovascular defects [21]. The *chd7* mutant zebrafish model effectively mimics the cardiovascular phenotype observed in CHARGE and non-syndromic patients with CHD, with the mutant zebrafish displaying abnormal first-branch arch branches similar to the clinical symptoms of patients with CHARGE syndrome [21]. Despite similarities in the initial formation and pattern of arch arteries between zebrafish and mammals, zebrafish do not undergo the same complex remodeling of the aorta and pulmonary arteries [20]. As many patients with CHARGE syndrome require surgical interventions for conditions, such as cardiovascular malformations and tracheoesophageal fistula, complications, including decreased oxygen saturation, respiratory rate, and abnormal heart rate, usually arise post-operatively [162]. Furthermore, these surgical interventions expose patients to increased doses of anesthesia and frequent experiences of adverse effects during anesthesia, such as an abnormal heart rate, reduced oxygen saturation, and decreased respiratory rate [163]. Observations show that *chd7* mutant zebrafish exhibit decreased heart rates, prolonged anesthesia requirements, and elevated respiratory rates upon awakening from anesthesia [164], and a study investigated the gene expression of phox2ba and phox2bb in a zebrafish model of CHARGE syndrome. As *PHOX2B* is involved in neural crest development, similar to *CHD7*, the loss of *PHOX2B* function can lead to respiratory issues resembling those in patients with CHARGE syndrome [149,164,165,166]. Leveraging the *chd7* mutant zebrafish model to simulate the anesthesia process in patients with CHARGE syndrome can deepen the understanding of the molecular underpinnings of these adverse events, enhancing the safety of anesthetic procedures in this population.

#### 3.2.2. Noonan Syndrome (NS)

Cardiac damage is a prominent NS feature, with CHDs occurring in 80% of patients [167]. The most prevalent manifestation of CHDs in NS is pulmonary valve stenosis (PVS), at approximately 40%, followed by atrial or ventricular septal defects (ASDs) and atrioventricular canal defects (AVCDs), at 8% and 15%, respectively [168]. *PTPN11* pathogenic variants are responsible for approximately 80% of cases involving PVS or ASDs [169]. Similarly, patients with AVCD and NS predominantly exhibit pathogenic variants in *PTPN11* [170]. Consequently, *PTPN11* emerges as a gene closely associated with NS-related CHDs. The (SHP2) protein encoded by *PTPN11* is distributed in the heart and regulates crucial processes, such as cell proliferation, migration, and differentiation during development [171]. In mouse *Ptpn11* knockout embryos, the embryos are damaged during implantation [172]. In zebrafish embryos, the complete absence of functional Shp2 allows for survival until 5–6 dpf; notably, the zebrafish genome encodes two *ptpn11* variants, *ptpn11a* and *ptpn11b*, and their respective encoded proteins Shp2a and Shp2b share 91% and 64% homology, respectively, with human SHP2 [173,174]. Further studies on zebrafish have revealed that double-knockout embryos for *ptpn11a* and *ptpn11b* exhibit severe cardiac edemas and craniofacial defects [68]. Creating the Shp2-D61G NS zebrafish model provides a specific disease target for different *shp2* mutants. Symptom variations in this mutant zebrafish model mirror those seen in individual human patients, with defects ranging in severity from mild to severe, including reduced body axis extension, heart edemas, craniofacial deformities, cardiac and mandibular edemas, significant developmental delays, and mortality before adulthood. Children with NS who have the D61G mutation are more likely to develop juvenile myelomonocytic leukemia-like myeloproliferative neoplasm (JMML-like MPN). Zebrafish embryos with the Shp2-D61G mutation also exhibited JMML-like MPN characteristics, including myeloid lineage expansion, mild anemia, and thrombocytopenia. Given the importance of fetal hematopoiesis in the development of JMML-like MPNs, the zebrafish Shp2-D61G mutant also serves as a dependable and distinctive model resembling JMML for studying the hematopoietic abnormalities resulting from SHP2 mutations in NS [69].

Shp2, a positive effector of Erk/mitogen-activated protein kinase (Mapk) signal transduction located downstream of most receptor tyrosine kinase (Rtks), is crucial in cellular processes [171,175]. The aberrant activation of the Mapk signaling pathway reportedly impairs ciliary function [176], particularly in NS, where pathogenic mutations in NS-related genes enhance this signaling cascade [177,178]. This emphasizes the significance of modulating Mapk signaling to prevent or ameliorate the onset of NS. One approach is the targeted inhibition of the rat sarcoma (Ras)/Mapk pathway, as demonstrated in a study that involved using trametinib, a MEK inhibitor, in infants with severe hypertrophic cardiomyopathy (HCM) and PVS associated with NS [179]. Notably, treatment with trametinib caused improvements in HCM and PVS, suggesting that the inhibition of Ras/Mapk overactivation could facilitate the remodeling of abnormal heart valves. Given the promising clinical outcomes observed with trametinib in NS, further systematic investigations are required to evaluate the impact of CHDs and their underlying biological mechanisms in NS. The zebrafish model is an attractive avenue for such studies because it can be rapidly used to assess the bioactivity and toxicity of various compounds using its absorbent gills and skin [180,181,182]. Zebrafish offer practical advantages over rodent models, including cost-effectiveness, ease of maintenance, and accelerated developmental processes, which can expedite study timelines and minimize animal stress induced by invasive procedures [183]. Trametinib has been less explored in zebrafish NS models, and existing studies primarily focus on its efficacy in zebrafish tumor models [184,185,186,187]. However, considering the benefits of zebrafish models and the clinical efficacy of trametinib, the zebrafish is a promising model system for investigating the intricate relationship between this drug and CHDs in NS.

#### 3.2.3. Alagille Syndrome (AGS)

AGS is an autosomal dominant, complex multisystem disorder with a clinical incidence rate of 1/100,000 live births [188,189]. TOF is the most common complex structural abnormality in AGS, occurring in up to 16% of cases [189,190]. Other malformations include ventricular septal defects, atrial septal defects, aortic stenosis, and aortic constriction, which significantly contribute to the high morbidity and mortality in patients with AGS [189]. AGS is commonly associated with the Notch signaling pathway [191], with most patients exhibiting pathogenic mutations in JAGGED1 (*JAG1)*-encoding Notch pathway ligands or in the receptor *NOTCH2* [73]. The existing AGS zebrafish model, generated by knocking down *jagged1b* and *jagged2b*, has primarily focused on studying liver pathology [70,192]. However, recent studies have shown that *jag1b*- and *jag2b-* knockout double mutants and jag2b knockout zebrafish develop cardiac edemas and liver abnormalities [70,71]. Although the cardiac defects in the AGS zebrafish model do not sufficiently replicate CHDs in human patients with AGS, the underlying molecular mechanisms causing heart defects are evolutionarily conserved. This finding offers valuable insights into the clinical treatment and pathogenesis of human-related diseases associated with AGS.

The ubiquitination of the Notch ligand intracellular tail and its subsequent activation, a process requiring Mind bomb 1 (Mib1) and Mib2 [193,194], is crucial for cellular signaling. Similarly, MIB1 is significant in the Wnt/β-catenin signaling pathway, influencing its regulation [195]. Knockout zebrafish lacking *mib1* exhibit cardiovascular abnormalities, including periaortic and intracranial hemorrhages and pericardial sac dilatation [72]. The significant differences in phenotypic characteristics between *mib1* knockout zebrafish and individuals with AGS question the fidelity of the zebrafish model in simulating AGS [73]. Despite this limitation, the study reveals the potential use of the zebrafish mutagenesis model for efficiently evaluating novel genetic variations, particularly in cardiovascular contexts. This model can facilitate the early identification of disease-causing factors, expediting CHD diagnosis and treatment [72].

#### 3.2.4. Axenfeld–Rieger Syndrome (ARS)

ARS is an autosomal dominant disorder characterized by ocular abnormalities. It also involves defects in cardiac development, such as mild left ventricular hypoplasia and abnormal valve and OFT formation [196,197,198]. The disease has an estimated prevalence of approximately 1 in 50,000 to 100,000 live births [199]. Approximately 40% of patients with ARS carry mutations in *FOXC1* or *PITX2* [200,201].

In zebrafish, there are two genes homologous to human FOXC1, namely *foxc1a* and *foxc1b*, with the proteins encoded by each sharing 66% and 55% homology with human FOXC1, respectively [202]. The genetic redundancy of *foxc1* in zebrafish has been previously reported [202]. When *foxc1a* is knocked out, juvenile fish exhibit various cardiovascular defects, including cardiac edemas, hypoplastic ventricles, shorter OFT, defective valve leaflets, and poor ventricular contractility, shortening their survival time [74,75]. These observed defects in zebrafish are similar to the clinical cardiac abnormalities in patients with ARS and *Foxc1* knockout mice [197]. Conversely, *foxc1b* knockout in zebrafish causes only a few juvenile fish to exhibit circulation defects, with no evident phenotype in adults [75,76,77]. Concurrent disruptions of both genes have been conducted subsequently, revealing that most double homozygous (*foxc1a* −/−; *foxc1b* −/−) zebrafish embryos develop heart defects, accompanied by more severe craniofacial malformations than those in *foxc1a* knockout homozygotes [75,203]. Furthermore, zebrafish with three mutant alleles (*foxc1a* +/−; *foxc1b* −/−) exhibit mild pericardial edemas and low survival rates, attributed to the thicker and denser myocardial zone in the fish, which impacts cardiac function [75,203]. Notably, other allele combinations do not exhibit visible phenotypes in embryos or adults. These findings suggest that *foxc1b* may compensate for *foxc1a* deficiency later in zebrafish heart development. When *foxc1b* is entirely lost, the normal phenotype cannot be maintained, underscoring the concept of genetic buffering by two paralogs. The evaluation of mutated genes in zebrafish embryos provides valuable insights into their potential roles in patients carrying the mutation, underscoring the significance of understanding the functional implications of genetic mutations in disease pathogenesis.

Recent studies have demonstrated that zebrafish *pitx2c* mutations can cause cardiac defects similar to those in individuals with ARS [204,205]. An investigation indicated that *pitx2c* mutant zebrafish larvae did not exhibit the full spectrum of cardiac abnormalities associated with ARS; however, it confirmed that the asymmetrical development of the heart tube and morphogenesis were unaffected by the mutation [206]. In contrast, another study focusing on adult zebrafish models with *pitx2c* loss-of-function mutations revealed the development of cardiac morphological irregularities, such as atrial dysplasia, increased fibrosis, arrhythmias, and fibrotic cardiomyopathy [78]. These findings reveal the cardiac phenotype observed in a subset of clinical patients with ARS, suggesting a potential association. However, these cardiac complications in adult fish may be a secondary effect of altered cell metabolism in the heart, an event that causes cardiac dysfunction [78]. Consequently, the collective findings underscore the significance of further investigating the role of metabolic pathways in ARS pathogenesis, leveraging the zebrafish mutation model as a valuable tool for future studies.

### 3.3. Teratogen-Induced Models of Embryonic Heart Development Defects

Approximately 80,000 synthetic chemicals are manufactured annually, with some of them being teratogens [207]. Compared to mammalian models (such as mice), zebrafish are an excellent model for studying the effects of teratogens on heart development, owing to their high fecundity, external embryo development, and independence from the mother during embryogenesis. In the following section, we discuss the impact of teratogens on zebrafish embryo heart development.

In pregnant women, diabetes increases the risk of congenital heart defects in the fetus by three to five times [208]. Hyperglycemia is a primary teratogenic condition that can affect the expression of genes essential for heart development [209]. In animal experiments, exposure to hyperglycemia can cause fetal malformations, similar to those observed in diabetic embryos, even when the mother does not have diabetes [210,211,212]. These studies support the view that hyperglycemia itself is teratogenic to embryos. Exposing zebrafish embryos to hyperglycemia results in a range of cardiac abnormalities, such as an increased incidence of ventricular stagnation and valve regurgitation, decreased peripheral blood flow, pericardial edemas, increased diameter and thinning of the heart wall, and the abnormal expression of tbx5a [210]. In another study, embryos treated with high glucose concentrations exhibited an abnormal tbx5 expression, pericardiac edemas, patent hearts, and valve regurgitation [213]. TBX5 mutation in humans can cause CHDs [214], and the deletion of tbx5 results in heart defects in zebrafish embryos, causing the swelling and relaxation of heart mitochondria [215]. TBX5 is possibly the main factor involved in fetal heart malformation induced by hyperglycemia.

Fish are highly susceptible to dioxin exposure, showing toxic effects, such as the cardiovascular dysfunction observed in other vertebrates [216]. Zebrafish eggs exposed to dioxins shortly after fertilization exhibited severe cardiac abnormalities in embryos, such as reduced cardiac output, diminished peripheral blood flow, and pericardial edemas [217,218,219]. The damage detected decreases as time after fertilization increases [217]. Adult zebrafish injected with a lethally high dose of dioxin did not exhibit any morphological changes in their hearts [220]. Research demonstrated that dioxins significantly influence embryonic development. Exposure to dioxins reduces the number of myocardial cells, as indicated by the expression of the marker gene *cmlc2* at 48 hpf [218]. Activating aryl hydrocarbon receptor 2 in zebrafish cardiomyocytes leads to the same defects, indicating that cardiomyocytes are the targets of dioxins [221].

Alcohol, the most widely used and consumed drug in daily human life, has a wide range of irreversible side effects on human fetuses. Maternal alcohol consumption is considered to have a high teratogenic effect on fetuses, affecting their development, including the heart. Research shows that in zebrafish embryos, exposure to ethanol can result in significant increases in cardiac edemas, decreases in cardiac volume, and reductions in ventricular thickness [222]. Alteration in the Bmp and Notch signaling pathways results in abnormal valve morphology [223]. During embryogenesis, the Bmp signal transduction pathway is essential for various stages of heart development, including cardiac specification, differentiation, endocardium differentiation, cardiac ring formation, chamber morphogenesis, and outflow tract separation [17,224,225,226,227]. The Notch signaling pathway is essential for endocardial differentiation and the formation of the OFT [228,229,230]. Folic acid has the potential to mitigate the damage caused by ethanol to embryonic heart morphology, although it does not fully remove it [231,232]. Folic acid’s antioxidant properties may weaken the oxidative stress caused by ethanol [233,234]. Thus, studying the impact of teratogens on the heart structure, morphology, and gene expression of zebrafish embryos, along with genetic techniques or drugs, can enhance our comprehension of disease origins and preventive strategies.

## 4. Future Directions

The zebrafish, a classic vertebrate with a high degree of genetic homology to humans, offers a valuable platform for examining the genes and mechanisms underlying CHDs. Using mature genetic and molecular technologies, researchers can efficiently screen and understand the genetic basis of human CHDs. Recognizing that certain pleiotropic gene mutations can cause numerous subclinical phenotypic effects is essential, as these events may collectively impact the fitness of affected individuals. Therefore, when specific disease candidate genes identified in patients with CHD are individually disrupted in zebrafish, corresponding phenotypes may manifest. In contrast, in humans, mutations in a single gene may not always cause observable effects and can involve multiple genes, highlighting the cumulative impact of pleiotropic genes. While the knockout of a single gene in zebrafish may not fully replicate the phenotypes in patients with heart disease-related syndromes, it may unveil evolutionarily conserved regulatory mechanisms governing vital developmental events in cardiac syndrome phenotypes. At the same time, human pluripotent stem cell models are used to corroborate findings obtained from the zebrafish model in investigating events, as the human genetic background can be preserved [235]. A further understanding of the defective phenotypes of gene deletions can offer insights into the origins of other phenotypes in humans, revealing potential treatment strategies for CHDs. Furthermore, by tracking cell lineage in regions affected by gene mutations associated with specific diseases, researchers can infer how genes influence other conditions during embryonic development. In recent years, several single nucleotide variants (SNVs) linked to CHDs have been discovered in humans, although the functions of many remain uncertain [236]. Preserving a substantial number of coding SNVs in zebrafish allows for quickly prioritizing disease variants in F0 and confirming their initial functional relevance in F1 [237]. The new base editing framework developed in this study is applicable to a wide range of SNV-susceptible traits in zebrafish, contributing to direct candidate validation and the prioritization of detailed mechanistic downstream studies [237]. The potential of zebrafish as a model for identifying the effects of SNV on cellular function during morphogenesis would be valuable. Leveraging the strengths of zebrafish in cardiac studies to establish a model for human CHDs can help elucidate the mechanisms underpinning these conditions and generate innovative approaches for their treatment and management.

## Figures and Tables

**Table 1:** Summary of advantages and disadvantages of zebrafish as a model for CHDs in vertebrates.

Advantages	Disadvantages
Similar to the electrophysiology of the human heart.	Absence of pulmonary circulation in the double-chambered heart.
Embryos can survive for a certain period even with impaired cardiovascular function.	Central venous pressure differs from that in humans.
Heart can regenerate after being injured.	The ventricles are primarily filled through atrial contraction.
Embryonic heart develops rapidly.	There are still differences in the ionic flow.
The formation pattern of the aortic arch is similar to that of mammals.	Lack of sarcolemmal T-tubules in cardiomyocytes.
Embryos and young fish are optically transparent.	Force–frequency relationship is distinct from that in humans.
The usability and accessibility of gene editing.	Lack a conduction system with specialized Purkinje fibers.
High homology with human genes.	Relatively limited antibodies and reagents.
Embryo development outside the body.	Fewer fibroblasts in adult fish hearts.
Ease, speed, and affordability of maintenance and breeding.	Genome duplication.

**Table 2:** Summary of zebrafish CHD model.

Simulated Human Disease	Human Gene Orthologs	Knock Zebrafish Gene	Zebrafish Main Phenotype	References
Heart chamber size defects	*NR2F1*	*nr2f1a*	The atria and atrioventricular canals are smaller.	[51]
	*SEMA3F*	*sema3fb*	The cardiac chambers become smaller; the cardiac muscle cells in the chamber become smaller.	[52]
Looping defects	*NODAL*	*spaw*	Abnormal cardiac looping.	[53,54]
	*DAND5*	*dand5*	Abnormal cardiac looping and displacement.	[53]
	*PKD2*	*pkd2*	Asymmetrical defects in the internal organs (including the heart) and brain.	[55]
	*WNT3a*	*wnt3a*	Cardiac asymmetrical defects.	[56]
	*HAS2*	*has2*	Displacement of the heart tube to the midline.	[57]
Atrioventricular valve defects	*KLF2*	*klf2a*	Valvular hypoplasia; absence of leaflets.	[58]
	*PKD1*	*pkd1a*	Increased incidence of retrograde blood flow.	[59]
	*TNNI1*	*tnni1b*	Phenotypes such as slow heart rate, lack of valve leaflets, and cardiac tube malformations.	[60]
	*NFATC1*	*nfatc1*	Atrial enlargement; retrograde blood flow in the atrioventricular canals.	[61,62]
Outflow tract stenosis	*TRAF7*	*traf7*	Embryonic heart displacement; pericardial edema and facial and cranial defects.	[63]
	*PAK1*	*pak1*	Outflow tract obstruction.	[64]
	*ACVRL1*	*acvrl1*	Outflow tract stenosis.	[65]
Tetralogy of Fallot	*BVES*	*bves*	Abnormal circularization; ventricular outflow tract stenosis.	[66]
	*WDR62*	*wdr62*	Abnormal circularization; narrowed heart chambers and thin walls.	[67]
CHARGE syndrome	*CHD7*	*chd7*	Abnormal branch of first branch arch.	[21]
Noonan syndrome	*PTPN11*	*ptpn11a*	Cardiac edema; craniofacial defects.	[68,69]
Alagille syndrome	*JAG1;AG2*	*jag1a;jag2b*	Cardiac edema.	[70,71]
	*MIB1*	*mib1*	Periaortic hemorrhage; cranial hemorrhage; and pericardial sac dilatation.	[72,73]
Axenfeld–Rieger syndrome	*FOXC1*	*foxc1a*	Cardiac edema; hypoplastic ventricles; short outflow tract; and poor ventricular contractility.	[74,75]
	*FOXC1*	*foxc1b*	Cardiac looping defect.	[75,76,77]
	*PITX2*	*pitx2*	Abnormal cardiac morphology; cardiac arrhythmia; and fibrosing cardiomyopathy.	[78]
